# Research on Resin Used for Impregnating Polyimide Fiber Paper-Based Composite Materials

**DOI:** 10.3390/ma14174909

**Published:** 2021-08-28

**Authors:** Wenlong Pang, Ruisen Shi, Jun Wang, Qingwei Ping, Xueru Sheng, Na Li, Jian Zhang

**Affiliations:** School of Light Industry and Chemical Engineering, Dalian Polytechnic University, Dalian 116034, China; pwl1106@163.com (W.P.); SRSshiruisen@163.com (R.S.); 18340800531@163.com (J.W.); lina@dlpu.edu.cn (N.L.); zhangjian@dlpu.edu.cn (J.Z.)

**Keywords:** polyimide fiber, modified, ternary resin

## Abstract

In this paper, a resin with high adhesion, easy curing, good flexibility, and high temperature resistance was prepared from polyimide fiber paper. First, in order to improve the toughness and curability of impregnating resin, epoxy resin was modified by addition of vinyl silicone resin. Subsequently, ternary resin with high temperature stability was obtained by polyimide resin addition. Among the investigated conditions, the optimal additive amount of vinyl silicone resin and polyimide resin was 30% and 5%, respectively. The prepared ternary resin has better toughness, crosslinking degree, high temperature stability (5% mass loss at 339.2 °C) and no obvious glass transition at high temperature. Finally, the polyimide fiber paper-based composite material was impregnated with modified epoxy resin and ternary resin, respectively. The results shows that the paper-based composite material impregnated with modified epoxy resin has a better fiber bonding degree, a smooth surface, and contact angle could reach up to 148.71°. Meanwhile, the paper-based composite material impregnated with ternary resin has good high temperature resistance, and the tensile index of the paper-based composite material could reach up to 35.1 N·m/g at 200 °C.

## 1. Introduction

Polyimide is a high-performance polymer material, and its main chemical structure is an imide ring with benzene ring and other five-or-six membered heterocyclic structures [1]. Due to the large bond energy and molecular chains of this distinctive structure, polyimide has relatively large intermolecular forces. Therefore, when the polyimide fiber is subjected to heat, high-energy radiation or external force, the absorbed energy can hardly the molecular chain, and the fiber exhibits excellent stability [2]. Compared with traditional high-performance fibers, such as aramid, P-Pphenylene-2,6-Benzoxazole (PBO) or polyethylene fiber [3,4], paper-based composite materials made of polyimide fiber have better high temperature resistance. Thus, much previous research has reported the preparation, characterization and applications of paper-based composite materials made of polyimide fiber [5].

Due to the lack of hydroxyl group, polyimide cannot participate in fibrillation, which leads to the poor mechanical property of the product [6]. Many different methods, such as blending [7], hot pressing [8], fiber modification [9,10,11], and impregnating [12,13], have been reported to improve the strength of paper-based composite materials made of polyimide fiber. Among these methods, impregnating possesses the advantage and several pioneer works have been reported. Gang et al. [14] used epoxy resin to impregnate polyimide fiber composites, which increased the mechanical properties of polyimide fiber paper-based composites by 1.25 times and the electrical properties by 17%. However, the temperature resistance of the composites was not perfect. The composites began to decompose at 350 °C (the decomposition temperature of original polyimide fiber was about 555 °C). Jiang Shufang et al. [15] reported the use of the silicone-modified epoxy resin during this process and the silicone group was introduced into the cross-linking network of the epoxy resin by formation of covalent bonds. Compared with the unmodified epoxy resin, the modified epoxy resin has a lower glass transition temperature and better mechanical properties [16]. More importantly, the epoxy resin was toughened significantly by the introduction of silicon–oxygen bonds, but the temperature resistance performance was not improved further. Stoica and others [17] reported a polyimide paper-based composite with good temperature resistance. They studied polyimide resin-reinforced paper-based composites from the perspectives of fiber distribution, pore size distribution, and form of reinforcement, but the mechanical properties of fiber-paper-based composites did not change significantly. The impregnating resins such as epoxy resin have very good adhesion. However, these resins suffer the problems of poor high temperature resistance [18]. Moreover, the brittleness of the product may also be aggravated after paper impregnation [19]. On the other hand, the modified epoxy resin has good thermal stability and toughness and the degree of crosslinking during curing can be also improved. The high temperature resistance is not outstanding, but that of polyimide resin is very good [20,21], though its disadvantage is poor adhesion and toughness. 

In this research, an innovative high-temperature resistant ternary resin was synthesized using silicone resin, epoxy resin, and polyimide resin as the main raw materials, and the polyimide fiber composite material was impregnated with this resin for the first time. The obtained composite material has a smooth surface, is permanently flame-retardant, has stable dimensions, and has a long service life, and can be used for high-efficiency smoke protection masks, fire protection and interior decoration of building materials.

## 2. Experimental Section

### 2.1. Materials and Chemicals

Polyimide fiber (length 4 mm), Jilin Changchun Gaoqi Polyimide Material Company Limited, Changchun, China; Aramid 1414 pulp, DuPont, Wilmington, DE, USA; epoxy resin (WSR6101), Nantong Xingchen Synthetic Material Company Limited, Nantong, China; vinyl silicone resin (DBR101), Wuhan Double Bond Chemical Sealing Material Company Limited, Wuhan, China; Silane coupling agent (KH560), a company in Guangdong, Guangdong, China; Polyimide resin (Industrial grade), Guangdong Wengjiang Reagent Chemical Company Limited, Guangdong, China; Dibutyltin dilaurate (Industrial grade), Shanghai Maclean Biochemical Technology Company Limited, Shanghai, China; N,N-Dimethylacetamide (AR), Sino pharm Chemical Reagent Company Limited, Beijing, China.; Imidazole (AR), Sinopharm Chemical Reagent Company Limited, Beijing, China; 4,4’-Diaminodiphenylsulfone (AR), Shanghai Maclean Biochemical Technology Company Limited, Shanghai, China.

### 2.2. Experimental Instrument

Kaiser Sheet Former (S001A), Beijing, China Pulp and Paper Research Institute Company Limited; Frank PTI Horizontal Tensile Machine (F81502), Frank Company, Berlin, Germany; Frank PTI Tearing Tester (F53984), Frank Company, Berlin, Germany; Thermal Reanalyzer (TGAQ500), Beijing Shenzhou Hengyi Technology Development Company Limited, Beijing, China; Fourier Transform Infrared Spectrometer (Frontier), PerkinElmer, Waltham, Massachusetts, United States; Differential Thermal Scanner (DISCOVERY), TA, Newcastle, PA, USA; Scanning Electron Microscope (JSM-6460LV), Hitachi, Tokyo, Japan; Contact angle measuring instrument (Theta), Bioolin, Gothenburg, Sweden.

### 2.3. Preparation of Modified Epoxy Resin

Dissolve vinyl silicone resin and epoxy resin in different mass ratios (the amount of vinyl silicone resin was 15%, 30%, 45%, 60% of the epoxy resin mass) in N,N-dimethylacetamide. Add silane coupling agent (5% of epoxy resin mass), dibutyltin dilaurate (0.5% epoxy mass), stirred and reacted for 2.5 h at 135 °C in a three-necked flask, and leave to stand after reaction is complete. The lower layer liquid obtains a modified epoxy resin, with 5% imidazole (calculated based on the solid content of the modified epoxy resin), 5% 4,4′-diaminodiphenyl sulfone (calculated based on the solid content of the modified epoxy resin), at 70 °C (1 h), 120 °C (3 h) for gradient heating curing.

### 2.4. Preparation of Ternary Resin

After diluting different quality polyimide resins (the amount of polyimide resin was 5%, 10%, 15%, and 20% of the modified epoxy resin mass) with N,N-dimethylacetamide, the modified epoxy resin was added and stirred at 120 °C for 0.5 h in a three-necked flask to obtain a ternary resin. Ternary resin uses 3% imidazole (calculated based on the solid content of the ternary resin) and 7% 4,4′-diaminodiphenyl sulfone (calculated based on the solid content of the ternary resin), at 70 °C (1 h), 120 °C (1 h), 180 °C (2 h) for curing with gradient heating.

### 2.5. Preparation of Polyimide Fiber Paper

The polyimide fiber and aramid pulp were wet-laid at a mass ratio of 7:3 to form a paper with a basis weight of 60 g/m^2^, and the paper was hot-pressed at 180 °C and 2.5 MPa for 1 min.

### 2.6. Paper-Based Composite Preparation

After diluting the impregnating resin to a solid content of 20% and adding a curing agent and a curing accelerator, the cut paper was placed into the resin for impregnation treatment, and then into an electric blast drying oven for curing to obtain a paper-based composite material.

### 2.7. Measurements

The chemical structure of the resin adopted PerkinElmer Fourier Infrared Spectrometer, the resin was evenly smeared on the pressed KBr blank sheet, and after it was completely dried, the structure was analyzed by Fourier Transform Infrared Spectrometer, at a wavenumber scanning range of 400–4000 cm^−1^.

The thermal stability of the resin was analyzed with a TGA Q500 thermogravimetric analyzer, the sample mass was 10 mg, the heating interval was 20 to 700 °C, the heating rate was 10 K/min, and the nitrogen flow rate was 40 mL/min. 

The morphology of the resin was observed using a JSM-6460LV scanning electron microscope. The analysis methods of the resin cross-section morphology were as follows: the prepared resin was poured into the mold and solidified; the specification was 40 mm × 20 mm × 5 mm. Then it was broken under the grip of a clamp, and the fractured surface was sprayed with gold and observed with a scanning electron microscope.

The tensile strength and elongation of the paper-based composite material was tested according to GB/T22898-2008, using a Frank PTI horizontal tensile machine, with a clamping distance of 50 mm, and cross-head speed of 10 mm/s. The length of the sample was 100 mm and the width 10 mm.

The surface properties of the paper-based composites were analyzed using a contact angle measuring instrument with 3D morphology analysis to discover the hydrophobic properties and smoothness of the surface of the paper-based composites.

## 3. Results and Discussions

Regarding the FT-IR analysis of the resin, due to the poor compatibility between silicon resin and epoxy resin [22], it is difficult to form a molecular-level uniform mixture by simple mechanical mixing. After a period of time, the stratification of mixture can be observed. To overcome this issue, vinyl silicone resin, instead of silicon resin, was introduced into the epoxy resin system through a chemical reaction and the compatibility of the mixture was improved.

**Figure 1:**
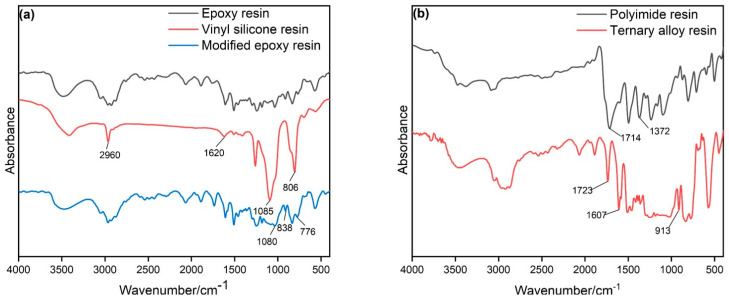
FT-IR spectrogram of different resins: (**a**) Modified epoxy resin (30% of silicone resin), (**b**) Ternary resin (5% of polyimide resin).

Figure 1 demonstrates the FT-IR spectrogram of different resins. In Figure 1a, the absorption peaks at 3500 cm^−1^, 1603 cm^−1^/1506 cm^−1^, and 913 cm^−1^ in epoxy resin were attributed to the –OH group, the vibration of benzene ring skeleton and the epoxy group, respectively. This spectrogram is consistent with the structural characteristics of bisphenol an epoxy resin [23]. For vinyl silicone resin, the absorption peaks at 3400 cm^−1^ indicate that there are some unreacted silanol hydroxyl groups in the silicone resin. At 2960 cm^−1^, the antisymmetric stretching vibration absorption peak of the saturated carbon atom C–H bond is reached. The absorption peak at 1620 cm^−1^ is the C=C bond stretching vibration absorption peak. The strong and broad peak appearing at 1080 cm^−1^ is the stretching vibration peak of the Si–O bond, and the strong peak appearing at 806 cm^−1^ is the stretching vibration of the Si–C bond. According to the above analysis, the obtained resin was a vinyl-terminated silicone resin with methyl as the side chain. The modified epoxy resin has a strong broad peak at 1085 cm^−1^, indicating that the Si–O bond is successfully connected to the epoxy resin. Meanwhile, the absorption peak at 1620 cm^−1^ of the vinyl silicone resin disappears, which may be due to the reaction of the double bond. During the reaction, the double bond was first opened and reacted with the hydroxyl group in the epoxy resin, followed by the formation of C–O–C bond. However, there was a large number of ether bonds in the epoxy resin, which led to the difficulty of attribution in the FT-IR spectrogram. The absorption peak at 913 cm^−1^ indicates that there are still many reactive epoxy groups in the modified epoxy resin [24].

For the polyimide resin (as shown in Figure 1b), the absorption peak at 1714 cm^−1^ comes from the symmetrical stretching of the C=O bond, and 1372 cm^−1^ is attributed to the stretching of the C–N bond [25]. It can be concluded that the polyimide resin used in this experiment has a higher degree of imidization, while, for the final obtained ternary resin, the symmetrical stretching vibration peak of the C=O bond at 1723 cm^−1^ and the characteristic absorption peak of the epoxy group at 913 cm^−1^ are still obvious, and there is no obvious new chemical bond formation [26]. The above indicates that the epoxy resin itself contains a large number of ether bonds. 

**Figure 2:**
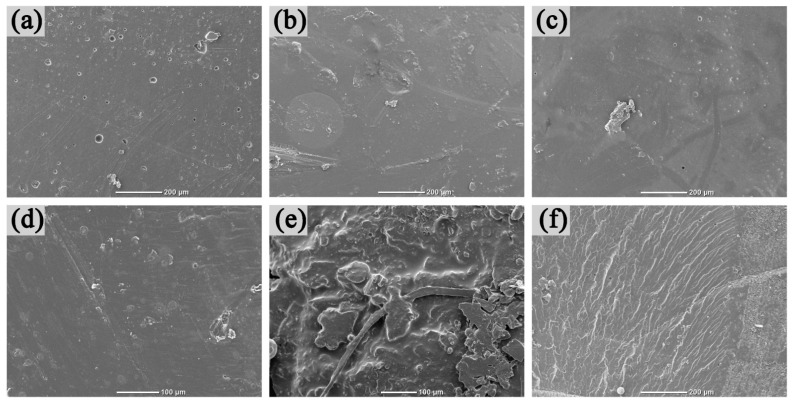
SEM topography of resin: (**a**) Epoxy surface, (**b**) Modified epoxy surface, (**c**) Ternary resin surface, (**d**) Epoxy fracture surface, (**e**) Modified epoxy resin fracture surface, (**f**) Ternary resin fracture surface.

Regarding the morphology analysis of the resin, Figure 2 is the SEM image. The amount of vinyl silicone resin in the modified epoxy resin was 30%, and the amount of polyimide resin in the ternary resin was 5%. As shown in Figure 2a–c, some porous structural defects can be observed after the curing of epoxy resin. After modification, the structural defects of the modified epoxy resin have been improved significantly, and the crosslinking has become more intense. In Figure 2d–f, the fracture surface of epoxy resin is very neat and exhibits obvious brittleness, while the resin modified by silicone resin becomes non-uniform and exhibits some void defect, indicating that the resin curing system absorbs more impact energy during the fracture process [27]. This can be mainly attributed to the Si–O bond in the molecular chain. This Si–O bond is easy to rotate, which can reduce the brittleness of the cross-linking system [16]. The fracture surface of the ternary resin is scaly and shows better uniformity.

**Figure 3:**
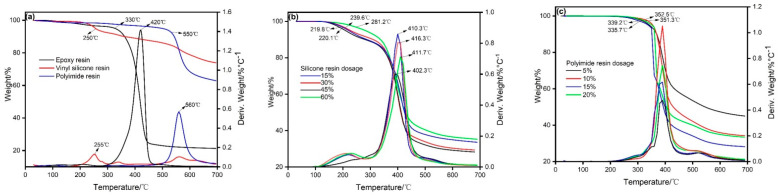
TGA analysis of resins: (**a**) Resin raw materials, (**b**) Modified resin raw materials, (**c**) Ternary resin.

Thermal properties of resin. Figure 3 illustrates the comparison of the thermal stability of resin raw materials, modified epoxy resins, and ternary resins. It can be seen from Figure 3a that the 5% mass loss temperatures of epoxy resin, vinyl silicone resin, and polyimide resin were 330 °C, 250 °C, and 500 °C, respectively, and the maximum mass loss rates were at 420 °C, 255 °C, and 560 °C. The carbon residue rates at 700 °C were 21.3%, 74.1% and 63.1%, respectively. Epoxy resin decomposes rapidly at high temperature, and the carbon residue rate is low, while the initial degradation temperature of vinyl silicone resin is low, and the carbon residue rate is high. This can be explained by the large amount of silicon and the high bond energy of silicon–oxygen bonds. Among investigated resins, polyimide resin has excellent heat resistance [25].

It can be seen from Figure 3b that, after modification with vinyl silicone resin, the 5% mass loss temperature decreases, which is related to the heat resistance of vinyl silicone resin itself. The difference is related to the significant increase in the carbon residue rate at 700 °C. As mentioned above, this is caused by the silicon element and the silicon–oxygen bond in the vinyl silicone resin. When the amount of vinyl silicone resin was 15%, 30%, 45%, and 60%, the 5% mass loss temperature of the modified epoxy resin was 219.8 °C, 239.6 °C, 220.1 °C, and 281.2 °C, respectively. The degradation rate of the Si–O bond decreased with the addition of chemical modification under the catalyst and high temperature conditions. Therefore, the 5% mass loss temperature of some modified epoxy resins was lower than the 5% mass loss temperature of the vinyl silicone resins. After curing, the quality loss, stall rate, and peak temperature of the modified epoxy resin system were 410.3 °C, 416.3 °C, 411.7 °C, and 402.3 °C, respectively. This change trend in heat resistance is related to two factors: (i) the addition of silicone chains can improve the mobility of molecules, so that the cross-linking degree of the resin system is closer after curing, thereby the heat resistance of the resin increased; (ii) vinyl silicone resin itself has relatively poor thermal stability, thus the thermal stability of resin decreased with excessive addition. In summary, the optimal dosage of vinyl silicone resin among investigated conditions was 30%.

It can be seen from Figure 3c that, when the amount of polyimide resin was 5%, 10%, 15%, and 20%, the 5% mass loss temperature of the ternary resin was 339.2 °C, 351.3 °C, 335.7 °C, and 352.7 °C. Compared with modified epoxy resin, the 5% mass loss temperature increased significantly, and the carbon residue rate also increased. However, compared with 5% dosage of polyimide resin, there was no obvious increase of 5% mass loss temperature of 20% dosage of polyimide resin. Thus, 5% of polyimide resin was chosen for economic consideration [25].

**Figure 4:**
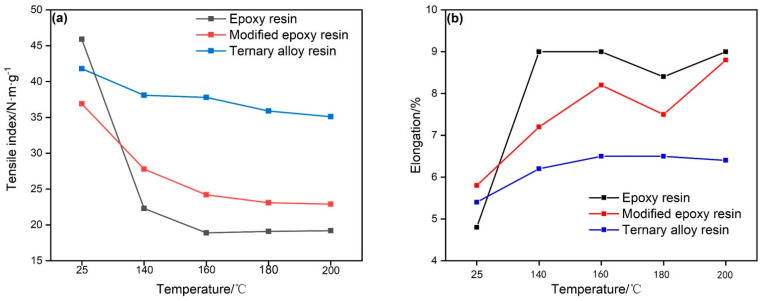
Performance comparison of different resin-impregnated paper-based composites: (**a**) Tensile strength, (**b**) Elongation.

Regarding the effect of resin impregnation on the strength properties of paper-based composites, when the amount of impregnated resin was 60% (relative to the absolute dry amount of the paper), the mechanical properties of different resin impregnated paper-based composites are shown in Figure 4. It can be seen that, after epoxy resin impregnation, the tensile index of the paper-based composite material reaches 45.9 N·m/g at room temperature, but the strength performance shows a certain downward trend with the increase of temperature. The tensile index of the modified epoxy resin impregnated paper-based composite material also changes significantly with temperature increase, due to the flexibility of the silicone resin molecular chain. As contrasted with the above materials, the tensile index of the paper-based composite material impregnated with ternary resin can be well maintained at high temperature. The tensile index of the paper-based composite material can reach 35.1 N·m/g at 200 °C and the breaking length of the paper-based composite material impregnated with ternary resin is lower.

**Figure 5:**
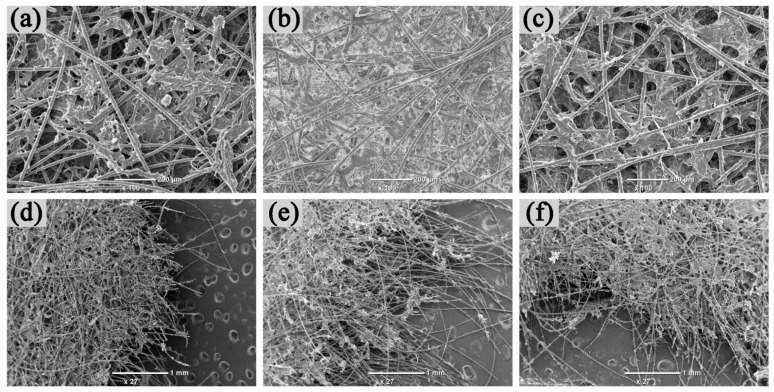
Surface and fracture surface morphology of different resin impregnated paper-based composites: (**a**) Epoxy impregnated material surface, (**b**) Modified epoxy resin impregnated material surface, (**c**) Ternary resin impregnated material surface, (**d**) Epoxy impregnated material fracture surface, (**e**) Modified epoxy resin impregnated material fracture surface, (**f**) Fracture surface of ternary resin impregnated material.

As for the morphology analysis of paper-based composites, the SEM images of the surface and the fracture of the paper-based composite are demonstrated in Figure 5. The resin impregnation amount is held at 60% (relative to the absolute dry amount of the paper). Compared with Figure 5a–c, it can be seen that the bonding degree between the modified epoxy resin and the fiber are better. On the contrary, the bonding degree between the other two resins was slightly worse. From Figure 5d–f, it can be seen that the fracture surfaces of these three resin-impregnated paper-based composites were not very neat, and many fibers were drawn out instead of being broken. This indicates that the strength of the paper-based composites depends on the strength of the paper-based composites. The bonding strength of the resin is the bonding strength between fibers.

**Figure 6:**
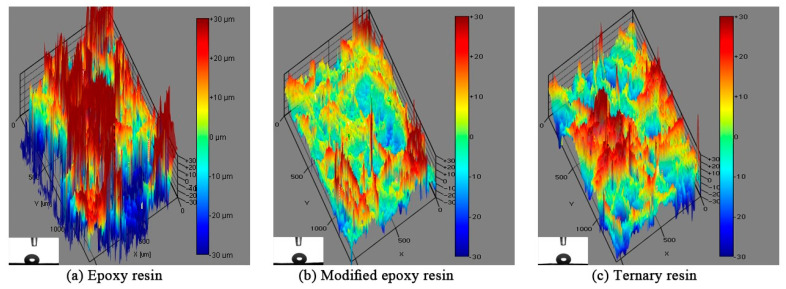
Surface morphology of paper-based composites impregnated with different resins: (**a**) Epoxy resin, (**b**) Modified epoxy resin, (**c**) Ternary resin.

**Figure 7:**
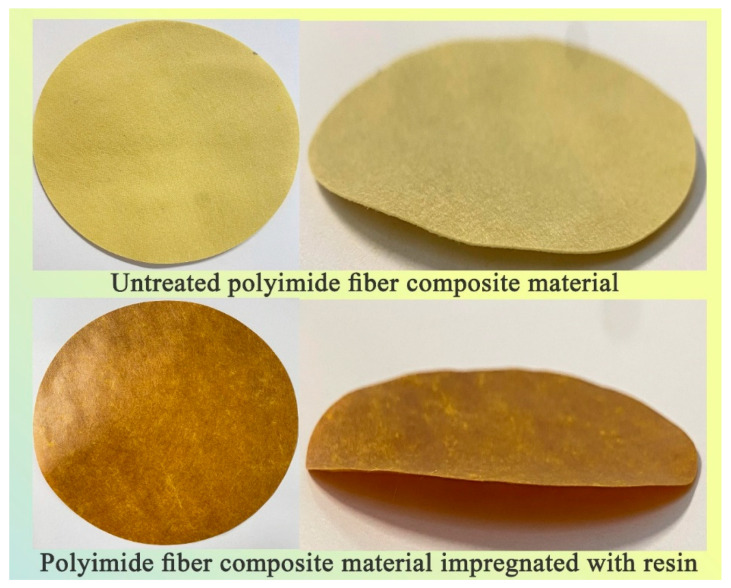
Polyimide fiber composite before and after work-up.

The surface morphology and contact angle of the paper-based composite materials are shown in Figure 6 and Figure 7. The resin impregnation amount was held at 60% (relative to the absolute dry amount of the paper). It can be seen from Figure 6 that the hydrophobicity of the surface of the modified epoxy resin impregnated paper-based composite improved significantly, and the surface of the paper-based composite was relatively flat. In addition, the surface contact angles of the epoxy resin, modified epoxy resin, and ternary resin impregnated paper-based composites were 117.13°, 148.71°, and 140.29°, respectively. This indicates that in the resin impregnated with silicone chains, the paper-based composite has good hydrophobic properties. The paper-based composite material impregnated with ternary resin also has lower surface energy, and the improvement of surface flatness needs further treatment.

## 4. Conclusions

This study focuses on the structure, morphology and thermal stability of epoxy resin, modified epoxy resin, and ternary resin, and the effect of impregnation on the properties of polyimide fiber paper-based composites (Its density is about 1.4 g/cm^3^, and its thickness does not exceed 0.6 mm).

1. The vinyl silicone resin modified epoxy resin forms a uniform system, the resin exhibits some void defects when it breaks, and its brittleness was lower than that of the unmodified epoxy resin. The ternary resin prepared by further adding of polyimide resin for the physical blending has good compatibility.
2. When the amount of vinyl silicone resin was 30%, the overall performance of the modified epoxy resin curing system was better, and the 5% mass loss temperature was 239.6 °C. The ternary resin was prepared with 5% polyimide resin, and the 5% mass loss temperature was increased to 339.2 °C. The starting point of the glass transition temperature of epoxy resin was 76.3 °C, and no obvious glass transition temperature was detected in the modified epoxy resin and ternary resin.
3. The epoxy resin-impregnated paper-based composite material has a tensile index of 45.9 N·m/g at 25 °C, but its strength decreases significantly at high temperatures. The effect of ternary resin on the high temperature resistance of paper-based composites is obvious. At 200 °C, the tensile index of paper-based composites can reach 35.1 N·m/g.
4. Paper-based composite materials impregnated with epoxy resin, modified epoxy resin and ternary resin were not broken when the fibers were broken, indicating that the strength of the paper-based composite material may be improved. Further processing was required. Among the three resins, the paper-based composite material impregnated with modified epoxy resin has a better fiber bonding degree, a smooth surface, a better hydrophobic effect, and a contact angle of 148.71°.

## Data Availability

Available upon request from the corresponding author.
